# An international multicentre analysis of current prescribing practices and shared decision-making in psoriatic arthritis

**DOI:** 10.1093/rheumatology/kead621

**Published:** 2023-11-27

**Authors:** Lily Watson, Conor Coyle, Caroline Whately-Smith, Melanie Brooke, Uta Kiltz, Ennio Lubrano, Rubén Queiro, David Trigos, Jan Brandt-Juergens, Ernest Choy, Salvatore D’Angelo, Andrea Delle Sedie, Emmanuelle Dernis, Sandrine Guis, Philip Helliwell, Pauline Ho, Axel J Hueber, Beatriz Joven, Michaela Koehm, Carlos Montilla, Jon Packham, José Antonio Pinto Tasende, Felipe Julio Ramirez Garcia, Adeline Ruyssen-Witrand, Rossana Scrivo, Sarah Twigg, Martin Soubrier, Théo Wirth, Laure Gossec, Laura C Coates

**Affiliations:** Department of Cellular and Molecular Medicine, University of Bristol, Bristol, UK; Oxford University Hospital, University of Oxford, Oxford, UK; Consultant Biostatistician, Whately-Smith Ltd, Herts, UK; Royal National Hospital for Rheumatic Diseases, Royal United Hospitals, Bath, UK; Rheumazentrum Ruhrgebiet, Herne, Germany; Ruhr-University Bochum, Herne, Germany; Academic Rheumatology Unit, Department of Medicine and Health Sciences “Vincenzo Tiberio”, University of Molise, Campobasso, Italy; Rheumatology and ISPA Translational Immunology Division, Hospital Universitario Central de Asturias, Oviedo-Asturias, Spain; Acción Psoriasis, Barcelona, Spain; Rheumatologische Schwerpunktpraxis, Berlin, Germany; CREATE Centre, Division of Infection and Immunity, Cardiff University, Cardiff, UK; Rheumatology Department of Lucania, San Carlo Hospital of Potenza, Potenza, Italy; Rheumatology Unit, Azienda Ospedaliero Universitaria Pisana, Pisa, Italy; Rheumatology Department, Centre Hospitalier du Mans, Le Mans, France; Rheumatology Department, CHU Marseille, Marseille, France; LIRMM, University of Leeds, Leeds, UK; Kellgren Centre for Rheumatology, Manchester University NHS Foundation Trust, Manchester, UK; Division of Rheumatology, Paracelsus Medical University, Klinikum Nürnberg, Nuremberg, Germany; Servicio de Reumatología, Hospital Universitario, 12 de Octubre, Madrid, Spain; Universidad Complutense de Madrid, Madrid, Spain; Division of Rheumatology, Goethe University, Frankfurt, Frankfurt am Main, Germany; Fraunhofer Institute for Translational Medicine and Pharmacology ITMP & Fraunhofer Centre of Excellence Immunemediated Diseaes CIMD, Frankfurt am Main, Germany; Rheumatology Department, Hospital Universitario Salamanca, Salamanca, Spain; Academic Unit of Population and Lifespan Sciences, University of Nottingham, Nottingham, UK; Rheumatology Department, Complexo Hospitalario Universitario A Coruña, Coruña, Spain; Arthritis Unit, Rheumatology Department, Hospital Clinic, Barcelona, Spain; Rheumatology Centre, Toulouse University Hospital, Toulouse, France; Paul Sabatier University, Toulouse III, Toulouse, France; Rheumatology Unit, Department of Clinical Internal, Anesthesiological and Cardiovascular Sciences, Sapienza University of Rome, Rome, Italy; Rheumatology, Bradford Teaching Hospitals NHS Foundation Trust, Bradford, UK; Rheumatology Department, CHU Clermont-Ferrand, Clermont-Ferrand, France; Rheumatology Department, CHU Marseille, Marseille, France; Sorbonne Université, INSERM, Institut Pierre Louis d’Epidémiologie et de Santé Publique, Paris, France; Rheumatology Department, AP-HP, Pitié-Salpêtrière Hospital, Paris, France; Nuffield Department of Orthopaedics, Rheumatology and Musculoskeletal Sciences, University of Oxford, Oxford, UK

**Keywords:** psoriatic arthritis, prescribing practices, shared decision-making, collaboration

## Abstract

**Objectives:**

Shared decision-making (SDM) is advocated to improve patient outcomes in PsA. We analysed current prescribing practices and the extent of SDM in PsA across Europe.

**Methods:**

The ASSIST study was a cross-sectional observational study of PsA patients ≥18 years of age attending face-to-face appointments between July 2021 and March 2022. Patient demographics, current treatment and treatment decisions were recorded. SDM was measured by the clinician’s effort to collaborate (CollaboRATE questionnaire) and patient communication confidence (PEPPI-5 tool).

**Results:**

A total of 503 patients were included from 24 centres across the UK, France, Germany, Italy and Spain. Physician- and patient-reported measures of disease activity were highest in the UK. Conventional synthetic DMARDs constituted a higher percentage of current PsA treatment in the UK than continental Europe (66.4% *vs* 44.9%), which differed from biologic DMARDs (36.4% *vs* 64.4%). Implementing treatment escalation was most common in the UK. CollaboRATE and PEPPI-5 scores were high across centres. Of 31 patients with low CollaboRATE scores (<4.5), no patients with low PsAID-12 scores (<5) had treatment escalation. However, of 465 patients with CollaboRATE scores ≥4.5, 59 patients with low PsAID-12 scores received treatment escalation.

**Conclusions:**

Higher rates of treatment escalation seen in the UK may be explained by higher disease activity and a younger cohort. High levels of collaboration in face-to-face PsA consultations suggests effective implementation of the SDM approach. Our data indicate that in patients with mild disease activity, only those with higher perceived collaboration underwent treatment escalation. Prospective studies should examine the impact of SDM on PsA patient outcomes.

**Trial registration:**

clinicaltrials.gov, NCT05171270.

Rheumatology key messages

- Disease characteristics and treatment strategies varied between countries, but particularly between the UK and mainland Europe.
- Patients reported high levels of SDM in face-to-face PsA consultations, unrelated to treatment escalation.
- Future analyses should examine the extent to which SDM influences patient outcomes in PsA.

## Introduction

PsA is a chronic inflammatory arthropathy affecting up to 30% of patients with psoriasis [1, 2]. The condition has a heterogeneous phenotype, with inflammation affecting the joints, tendons, soft tissue, skin, nails and spine to differing extents between patients. PsA is associated with a reduced life expectancy and significant impact on quality of life through musculoskeletal symptoms and associated comorbidities [3, 4]. Multiple pharmacological treatment options exist, with significant developments in the field of DMARDs in recent years [5]. There has been an expansion of treatment options beyond traditional conventional synthetic DMARDs (csDMARDs), such as methotrexate, sulfasalazine and leflunomide, to targeted therapies including biologic DMARDs (bDMARDs) that target underlying pathogenic molecules, such as tumour necrosis factor (TNF), IL-12/23, IL-23 and IL-17A/F and targeted synthetic DMARDs such as Janus kinase (JAK) and phosphodiesterase-4 (PDE4). National and international guidelines have been developed to inform treatment decisions in PsA [6, 7]. However, treatment decisions must be tailored to the individual, given the heterogeneity in clinical phenotype and the varying efficacy of each treatment in different disease domains. Treatment approaches are likely to vary by geographical location, driven by differences in healthcare services and reimbursement, but an observational analysis of current prescribing practices in PsA has not been undertaken.

Shared decision-making (SDM) is an important component of personalized healthcare, where treatment selection is guided by collaboration between the clinician and the patient to ensure the incorporation of patient priorities and values. SDM has been shown to improve outcomes and increase treatment compliance across multiple clinical groups [8–11]. It relies on both the clinician’s effort to incorporate patient priorities and the patient’s confidence in voicing their values. The CollaboRATE questionnaire and 5-item Perceived Efficacy in Patient-Physician Interactions (PEPPI-5) tool are patient-reported measures of the clinician’s collaborative effort and patient communication confidence, respectively [12–15] (Supplementary Table S1, available at *Rheumatology* online). Despite SDM being a top international priority, there are no studies examining the degree of SDM in PsA consultations to date [16].

We undertook an international observational analysis to examine and compare current prescribing practices and SDM in PsA consultations across Europe.

## Methods

### Study population and design

A subanalysis was undertaken using data from the international, cross-sectional ASSIST study to explore treatment decisions and SDM in adult PsA consultations across Europe. Ethical approval was received for this research study via research ethics committee (reference 20/PR/0587) and has been listed via the Integrated Research Application System platform (ID: 287039). Patients ≥18 years of age attending a face-to-face rheumatology appointment at a specialist rheumatology centre between July 2021 and March 2022 were eligible for inclusion (NCT05171270). All patients had to have previously received a diagnosis of PsA by a rheumatologist according to the Classification Criteria for Psoriatic Arthritis [17]. Patients were selected by systematic sampling, with a different random starting patient ‘number’ per centre, from 24 centres across the UK, France, Germany, Italy and Spain. The target was 100 patients per country and at least 15 per centre. Patients gave written informed consent to participate. Patients were not eligible for the study if they had a new diagnosis of PsA at the current clinic visit, were not comfortable completing an app-based questionnaire or paper case-report form or were unable to speak/read the local language.

### Data

The following patient and disease characteristics were recorded: patient demographics, PsA duration, current treatment, number of comorbidities (according to the functional comorbidity index [18]) and disease activity. Disease components were measured by:

- A clinical assessment of tender and swollen joint counts, dactylitis count, body surface area of psoriasis and physician numerical rating score (NRS) of overall disease activity
- Leeds Enthesitis Index (LEI) [19]
- 12-item Psoriatic Arthritis Impact of Disease (PsAID-12) questionnaire via the Group for Research and Assessment of Psoriasis and Psoriatic Arthritis (GRAPPA) app on a tablet (scored from 0 to 10, with 10 reflecting worst possible health) [20]. The PsAID-12 score is a weighted sum of the scores for the 12 questions divided by 20.
- Patient NRS for global disease activity (psoriasis and arthritis) and pain (scored from 0 to 10, with 10 reflecting the highest disease activity) [21]
- The HAQ, scored 0–3 with 3 being worst health [22]
- The European Quality of Life 5-Dimensions questionnaire (EQ-5D) visual analogue scale (VAS) for current health (scored 0–100, with 100 being the best possible health) [23].

Treatment decisions were documented as no change in treatment, treatment escalation or treatment reduction. Treatment escalation included a dose increase, frequency increase, altered route of administration, medication addition or medication switch. The three-item patient-reported CollaboRATE questionnaire and PEPPI-5 tool were completed at the end of consultations, independent from clinicians [12, 13] (Supplementary Table S1, available at *Rheumatology* online). The mean CollaboRATE score (mean score across three items) was recorded per patient. As per the CollaboRATE scoring manual, two measures were calculated for each patient: the mean score for the three questions and a simple yes/no outcome if all three items were scored as the maximum of 9. The recorded PEPPI-5 score per patient is the sum of scores for the five items.

### Statistical analysis

Descriptive statistics were calculated for patient demographics, disease activity, prescribing practices, CollaboRATE and PEPPI-5 scores across countries. For PEPPI-5 analysis, the mean of the recorded score (sum of the five items) was calculated per country and in male and female patients. Normally distributed data with a low number of outliers is represented by mean and s.d. Skewed data or those with significant outliers are represented by median and interquartile range (IQR)/minimum–maximum. Boxplots were created for the physician NRS, PsAID-12, HAQ, CollaboRATE and PEPPI-5 scores by country. Returned CollaboRATE or PEPPI-5 questionnaires missing one or more responses were excluded from analysis.

## Results

### Demographic and clinical characteristics

**Figure 1.**
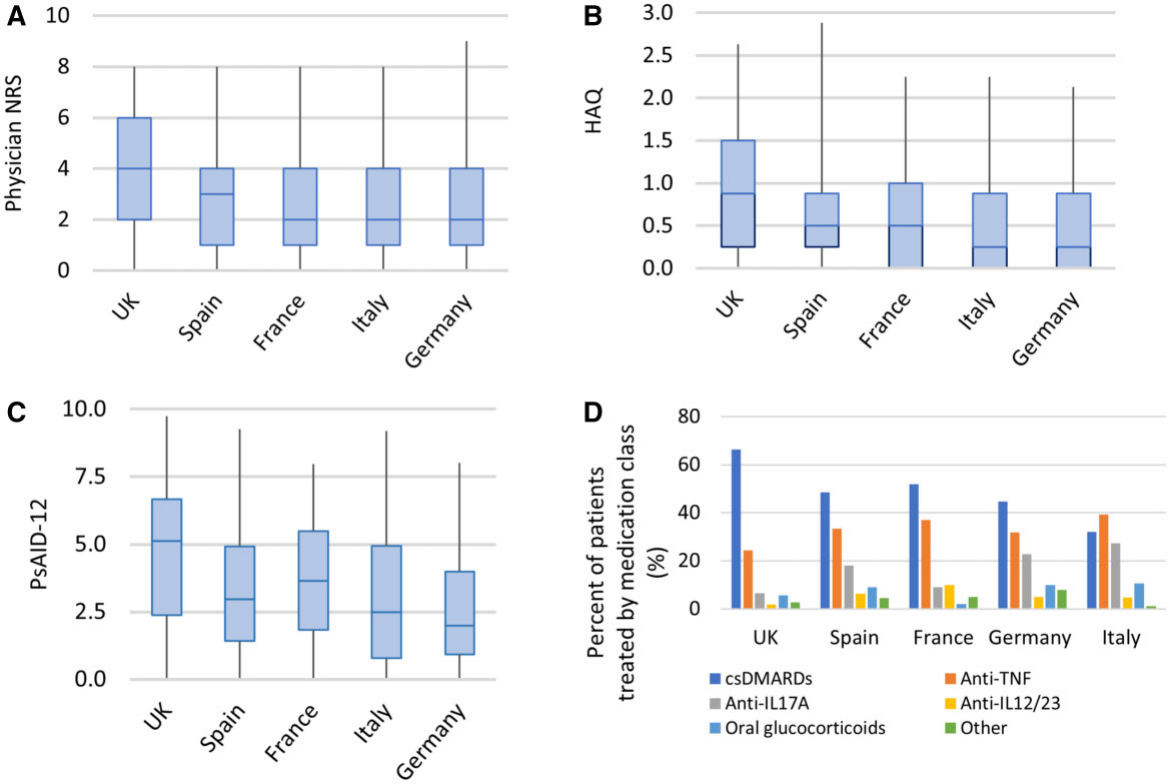
Box plots of disease activity/physical function and bar chart of medication class by country. (**A**) Physician NRS of overall global assessment (scored 0–10, with 10 as worst disease), (**B**) HAQ (scored 0–3, with 3 as worst health), (**C**) PsAID-12 score (scored 0–10, with 10 as worst disease) and (**D**) medication class by country

A total of 503 patients were recruited from 24 centres across the UK, Spain, France, Italy and Germany. Patient demographics varied by country (Table 1). Overall, 247 (49.1%) patients were female and mean patient age was 53 years (range 18–83). The UK had the lowest mean patient age, while Italy and France had a notably higher proportion of males. In all countries, the most common PsA subtype was peripheral arthritis (83.7% of all patients) and the number of comorbidities was low. Patient and physician markers of disease activity reflected mild disease across all countries (Table 1, Fig. 1), although median scores for tender joint counts, patient-reported and physician-reported disease activity, PsAID-12, EQ-5D VAS and HAQ were highest in the UK. The extent of missing data was low. Missing data was most common for the number of comorbidities (18 patients, 3.6% of the study population). PsAID-12 data was available for all but two patients.

**Table 1.** Patient demographics and disease characteristics by country

Characteristics	France (*n* = 100)^a^	Germany (*n* = 101)	Italy (*n* = 84)	Spain (*n* = 111)	UK (*n* = 107)	All (*N* = 503)
Centres, *n*	5	5	4	5	5	24
Age, years, mean (s.d.)	54.9 (12.4)	55.3 (12.1)	54.3 (11.7)	53.8 (11.5)	51.6 (13.6)	53.9 (12.3)
Female, *n* (%)	47 (47.0)	58 (57.4)	29 (34.5)	54 (48.6)	59 (55.1)	247 (49.1)
Comorbidities, median (minimum–maximum)	1.0 (0–7)	1.0 (0–7)	1.0 (0–4)	1.0 (0–7)	1.0 (0–11)	1.0 (0–11)
Disease duration, years, mean (s.d.)	12.8 (9.6)	9.0 (8.5)	11.8 (11.2)	11.0 (8.7)	9.7 (8.3)	10.8 (9.3)
Disease subtype, *n* (%)						
Peripheral arthritis	72 (72.0)	84 (83.2)	73 (86.9)	91 (82.0)	101 (94.4)	421 (83.7)
Axial	21 (21.0)	11 (10.9)	5 (6.0)	15 (13.5)	4 (3.7)	56 (11.1)
Enthesitis	7 (7.0)	6 (5.9)	6 (7.1)	4 (3.6)	1 (0.9)	24 (4.8)
Tender joint count, median (IQR)	1.0 (0.0–4.0)	0.0 (0.0–3.0)	2.0 (0.0–5.0)	1.0 (0.0–4.0)	3.0 (0.0–9.0)	2.0 (0.0–4.0)
Swollen joint count, median (IQR)	0.0 (0.0–0.0)	0.0 (0.0–0.0)	0.0 (0.0–1.0)	0.0 (0.0–2.0)	1.0 (0.0–3.0)	0.0 (0.0–1.0)
Dactylitis count, median (IQR)	0.0 (0.0–0.0)	0.0 (0.0–0.0)	0.0 (0.0–0.0)	0.0 (0.0–0.0)	0.0 (0.0–0.0)	0.0 (0.0–0.0)
Psoriasis body surface area, *n* (%)						
Clear	37 (37.0)	39 (38.6)	28 (33.3)	37 (33.3)	34 (31.8)	175 (34.8)
≤3%	54 (54.0)	60 (59.4)	39 (46.4)	71 (64.0)	63 (58.9)	287 (57.1)
>3%	9 (9.0)	2 (2.0)	17 (20.3)	3 (2.7)	10 (9.3)	41 (8.2)
Leeds Enthesitis Score, *n* (%)						
0	70 (70.0)	86 (85.1)	54 (64.3)	81 (73.0)	68 (63.6)	359 (71.4)
1	5 (5.0)	5 (5.0)	10 (11.9)	7 (6.3)	12 (11.2)	39 (7.8)
2	15 (15.0)	6 (5.9)	7 (8.3)	10 (9.0)	12 (11.2)	50 (9.9)
≥3	8 (8.0)	2 (2.0)	15 (17.9)	7 (6.3)	10 (9.3)	40 (7.9)
Physician NRS of overall disease activity, median (IQR)	2.0 (1.0–4.0)	2.0 (1.0–4.0)	2.0 (1.0–4.0)	3.0 (1.0–4.0)	4.0 (2.0–6.0)	3.0 (1.0–5.0)
Patient NRS of global disease activity, median (IQR)	3.7 (1.9–5.8)	2.1 (0.9–4.3)	2.6 (0.9–4.9)	3.3 (1.6–5.2)	5.4 (2.7–6.9)	3.5 (1.5–5.7)
Patient NRS of pain, median (IQR)	4.0 (2.0–6.5)	3.5 (1.5–6.0)	3.0 (1.0–7.0)	4.0 (2.0–6.5)	6.0 (3.0–7.5)	4.0 (2.0–7.0)
PsAID-12 score, median (IQR)	3.7 (1.8–5.5)	2.0 (0.9–4.0)	2.5 (0.8–5.0)	3.1 (1.5–5.0)	5.1 (2.4–6.7)	3.3 (1.3–5.4)
HAQ, median (IQR)	0.5 (0.0–1.0)	0.3 (0.0–0.9)	0.3 (0.0–0.9)	0.5 (0.3–0.9)	0.9 (0.3–1.5)	0.5 (0.0–1.0)
EQ-5D VAS for current health, median (IQR)	60 (50.0–80.0)	70.0 (42.5–85.0)	70.0 (50.0–80.0)	70.0 (55.0–80.0)	60.0 (40.0–75.0)	65.0 (50.0–80.0)

a *n* = *x* in the column headers refers to the total study population in each country and may vary between the outcomes summarized due to different missing data patterns.

### Prescribing practices

Current prescribing practices varied by country (Table 2). The use of glucocorticoids was uncommon across countries. csDMARDs formed the predominant treatment in the UK (66.4% of UK patients) but were less frequently used in continental Europe (32.1–52.0% of patients per country). Conversely, bDMARDs were the most frequently used medication in all countries other than the UK. Among csDMARDs, the most commonly used medication in all countries was methotrexate (38.6% of all patients). The preference for bDMARD varied: adalimumab was the most used bDMARD in the UK and Spain, adalimumab and secukinumab were equally used in Germany and ixekizumab and adalimumab were joint-first in Italy.

**Table 2.** Current prescribing practices and treatment decisions by country^a^

Characteristics	France (*n* = 100)^b^	Germany (*n* = 101)	Italy (*n* = 84)	Spain (*n* = 111)	UK (*n* = 107)	All (*N* = 503)
Any csDMARD, *n* (%)	52 (52.0)	45 (44.6)	27 (32.1)	54 (48.6)	71 (66.4)	249 (49.5)
Methotrexate	43 (43.0)	38 (37.6)	23 (27.4)	40 (36.0)	50 (46.7)	194 (38.6)
Leflunomide	3 (3.0)	3 (3.0)	1 (1.2)	6 (5.4)	4 (3.7)	17 (3.4)
Sulfasalazine	0 (0.0)	0 (0.0)	4 (4.8)	6 (5.4)	19 (17.8)	29 (5.8)
Other	4 (4.0)	3 (3.0)	2 (2.4)	2 (1.8)	5 (4.7)	16 (3.2)
Any bDMARD, *n* (%)	63 (63.0)	69 (68.3)	62 (73.8)	69 (62.2)	39 (36.4)	302 (60.0)
Anti-TNF	37 (37.0)	32 (31.7)	33 (39.2)	37 (33.3)	26 (24.3)	165 (32.8)
Anti-IL-17A	9 (9.0)	23 (22.8)	23 (27.4)	20 (18.0)	7 (6.5)	82 (16.3)
Anti-IL-12/23	10 (10.0)	5 (5.0)	4 (4.8)	7 (6.3)	2 (1.9)	28 (5.6)
Other	5 (5.0)	8 (7.9)	1 (1.2)	5 (4.5)	3 (2.8)	22 (4.4)
Oral glucocorticoids, *n* (%)	2 (2.0)	10 (9.9)	9 (10.7)	10 (9.0)	6 (5.6)	37 (7.4)
Treatment decisions, *n* (%)						
No change in treatment	70 (70.0)	67 (66.3)	60 (71.4)	72 (64.9)	52 (48.6)	321 (63.8)
Treatment increase	28 (28.0)	26 (25.7)	20 (23.8)	35 (31.5)	51 (47.7)	160 (31.8)
Treatment decrease	2 (2.0)	8 (7.9)	4 (4.8)	4 (3.6)	4 (3.7)	22 (4.4)
Treatment increase, *n* (%)						
Dose	8 (8.0)	4 (4.0)	3 (3.6)	7 (6.3)	8 (7.5)	30 (6.0)
Frequency	3 (3.0)	4 (4.0)	0 (0.0)	3 (2.7)	1 (0.9)	11 (2.2)
Route change	1 (1.0)	0 (0.0)	0 (0.0)	4 (3.6)	1 (0.9)	6 (1.2)
Additional medication	9 (9.0)	12 (11.9)	6 (7.1)	16 (14.4)	28 (26.2)	71 (14.1)
Replacement medication	8 (8.0)	9 (8.9)	13 (15.5)	9 (8.1)	15 (14.0)	54 (10.7)

a Note that patients could have had more than one reason for and increase/decrease, so percentages may not sum to 100.

b *n* = *x* in the column headers refers to the total study population in each country and may vary between the outcomes summarized due to different missing data patterns.

A decision to alter the current treatment regime occurred in 36.2% (182 patients) of the cohort, with treatment escalation being the predominant change (160 patients) (Table 3). Only 22 patients (4.4%) had their treatment decreased after their consultation. Notably, the frequency of treatment escalation was highest in the UK, occurring in nearly half of all UK consultations [51 patients (47.7%)], and lower in continental Europe (ranging from 23.8 to 31.5% per country). The predominant method to achieve treatment escalation was medication addition in all countries except Italy, where medication switch was most common.

**Table 3.** CollaboRATE and PEPPI-5 scores by country

Score	France (*n* = 100)^a^	Germany (*n* = 101)	Italy (*n* = 84)	Spain (*n* = 111)	UK (*n* = 107)	All (*N* = 503)
CollaboRATE mean score, median (IQR)	9.0 (8.3–9.0)	9.0 (8.0–9.0)	7.3 (5.7–8.3)	8.3 (7.3–9.0)	9.0 (8.7–9.0)	9.0 (7.7–9.0)
CollaboRATE maximum score, *n* (%)	70 (70.0)	55 (57.3)	21 (25.0)	49 (44.1)	71 (67.6)	266 (53.6)
PEPPI-5 total score, median (IQR)	25.0 (21.0–25.0)	22.0 (20.0–25.0)	20.0 (17.0–23.0)	22.0 (18.0–25.0)	25.0 (21.0–25.0)	23.0 (20.0–25.0)

a *n* = *x* in the column headers refers to the total study population in each country and may vary between the outcomes summarized due to different missing data patterns.

### SDM

**Figure 2.**
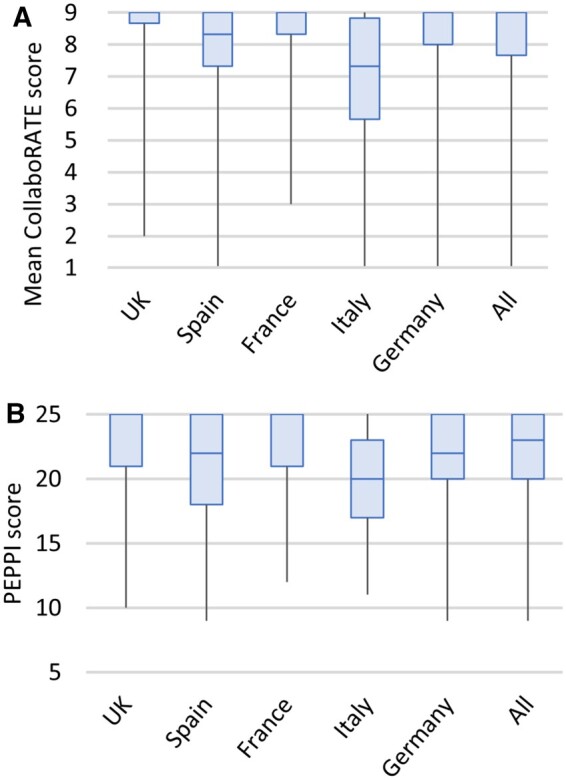
Box plots by country of (**A**) mean CollaboRATE score and (**B**) PEPPI-5 score

Completed CollaboRATE and PEPPI-5 questionnaires were collected from 496 and 494 patients, respectively (incomplete or unreturned questionnaires from 7 and 9 patients, respectively). CollaboRATE and PEPPI-5 scores were positively skewed to the upper limit (Fig. 2, Table 3). Of all completed questionnaires, 53.6% had the highest possible CollaboRATE score. The total PEPPI-5 scores were similar for males and females (mean = 21.4 for both), as were the CollaboRATE scores (mean = 8.0 for both). When comparing patients with the lowest 5% of PEPPI-5 scores with the remaining cohort, the mean age (53.4 *vs* 53.9 years), percentage of female patients (60.0% *vs* 48.2%) and mean disease duration (11.3 *vs* 10.8 years) were similar.

**Figure 3.**
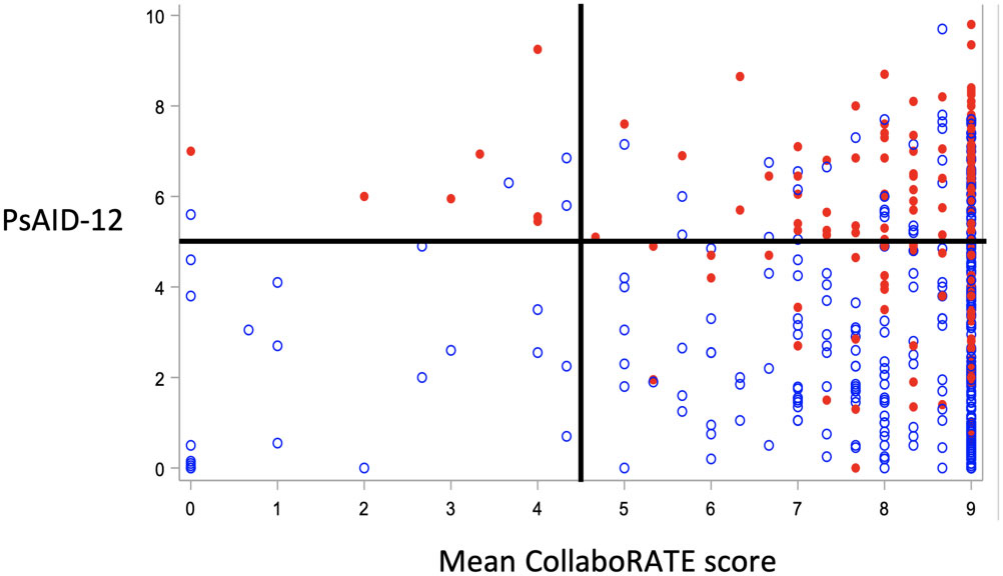
Treatment escalation (red) *vs* no escalation (blue) according to mean CollaboRATE and PsAID-12 scores per patient

There was no clear association between treatment escalation and CollaboRATE or PEPPI-5 scores: the mean CollaboRATE and PEPPI-5 scores were similar in those with and without treatment escalation (mean CollaboRATE 8.1 *vs* 7.9, mean PEPPI-5 21.3 *vs* 21.5) and the percentage of patients providing a maximum CollaboRATE score was similar irrespective of treatment escalation or not (51.9% *vs* 53.4%). The relationship between CollaboRATE, PsAID-12 and treatment escalation was examined (Fig. 3). Of 31 patients with a low CollaboRATE score (<4.5), treatment escalation only occurred in patients with a PsAID-12 score ≥5 and not in any patients with PsAID-12 score <5. In contrast, of 226 patients with CollaboRATE scores ≥4.5, treatment escalation occurred in patients with high and low PsAID-12 scores, including 59 patients with a PsAID-12 score <5.

## Discussion

The PsA cohort is highly heterogeneous in clinical phenotype and treatment responsiveness, making treatment decisions complex and multifactorial [5]. Current prescribing practices are likely to vary by geographical location, but this has not been well described. In this multicentre international analysis of routine clinical practice, we found significant variation in prescribing practices by country. Notably, the frequency of bDMARD use in continental Europe was significantly higher than the UK, where csDMARD use predominated. National Institute for Health and Care Excellence guidelines advise the use of at least two csDMARDs prior to starting biologic therapy in the UK and requires patients to have at least three tender and swollen joints, explaining the predominance of csDMARDs in the UK cohort [24]. In Europe, guidelines vary by country, but the use of biologics is not generally restricted to a number of affected joints/enthesitis or a prerequisite of failing two DMARDs [25–27]. The more frequent use of methotrexate in the UK may also relate in part to the greater predominance of peripheral arthritis in the UK cohort. The level of disease activity across multiple patient- and physician-reported outcome measures was highest in the UK, potentially reflecting current prescribing differences or a selection bias. The UK has 25% fewer physicians per 1000 people than mainland Europe [28]. Post-coronavirus disease 2019 clinical pressures in the UK during ASSIST recruitment meant that those attending a face-to-face consultation were those with active disease flares. A higher capacity to review stable/non-flaring patients in Europe may explain some of the geographic differences in disease severity and treatment choices between the UK and mainland Europe. Treatment escalation was more common in the UK (47.7% of patients) than Europe (23.8–31.5% of patients per country), in keeping with the higher level of physician- and patient-reported disease activity, the predominance of csDMARD use and the younger patient demographic in the UK, with treatment escalation being more likely earlier in the disease course.

SDM is crucial in PsA, given the variation of clinical phenotypes and treatment efficacies in different disease domains. SDM can also help overcome the discordance in assessment of disease activity by the patient and the clinician, which is particularly noted in mild disease [29]. Despite its importance, the extent of SDM in PsA has not been examined as of yet. We measured clinician collaborative effort and patient communication confidence with CollaboRATE and PEPPI-5 questionnaires [12, 13]. Reassuringly, we found high CollaboRATE and PEPPI-5 scores across centres, irrespective of treatment decision. Patients were recruited by systematic sampling with a random starting number to minimize selection bias and questionnaires were completed independent from clinicians. These results differ from previous analyses of SDM that reported lower rates of SDM in other inflammatory arthropathies. A self-reported analysis of SDM among rheumatologists treating RA in Japan found only 27% practiced SDM and an independent observational analysis of recorded RA consultations in the Netherlands found a mean score of 28/100 on the observer patient involvement scale, an alternative measure of SDM [30, 31]. The contrast with our findings may reflect an increased awareness of and/or training in SDM in recent years. However, our findings may have been skewed by the Hawthorne effect (where the knowledge of SDM examination may have influenced the usual practice of physicians).

In consultations with lower levels of reported collaboration, treatment escalation was only seen in patients with higher disease impact (PsAID-12 score >5). However, in consultations with high levels of clinician effort to collaborate, treatment escalation occurred in patients with mild or active disease (low or high PsAID-12 scores). This may reflect improved identification of symptoms/concerns in more collaborative consultations that subsequently justify treatment escalation, underlining the importance of SDM. However, the data could be explained by retrospective bias, where patients who receive treatment escalation are more likely to report their consultation as collaborative than those who did not.

Data generalizability was enhanced by undertaking an international analysis of >500 participants, including multiple centres per country. However, all patients were recruited from specialist PsA clinics and disease activity was generally low, which may differ from other rheumatology clinics. Limitations of our study also include clinician awareness of SDM assessment, the ineligibility of patients who were unable to speak/read the local language and inclusion of only face-to-face consultations. The latter two factors may have introduced biases given the probability of higher levels of disease activity within these groups. Making valid international comparisons is not without challenges [32]. To overcome these, consistent and accurate data measurement was optimized with a thorough protocol and use of questionnaires that have been internationally validated. Nevertheless, cultural variation may influence the subjective nature of perceived collaboration and patient confidence. With an increasing frequency of virtual consultations, it is important to assess whether high levels of SDM are maintained on online platforms. Future prospective research should examine whether higher levels of SDM translate into improved PsA outcomes and qualitative work is needed to identify factors associated with more collaborative consultations. This may help guide further improvements in the practice of SDM.

## Conclusion

This study delineates current PsA prescribing practices, disease characteristics and SDM across multiple centres in the UK, France, Germany, Italy and Spain. Disease characteristics and treatment strategies varied between countries, but particularly between the UK and mainland Europe. In keeping with a greater restriction on bDMARD use, csDMARDs predominated in the UK. Patients reported high levels of SDM in face-to-face PsA consultations, unrelated to treatment escalation. In patients with low PsAID-12 scores, those with higher perceived collaboration were more likely to have treatment escalation than those without. Further qualitative analyses are needed to understand the rationale for treatment escalation in cases with lower disease activity and examine the extent to which SDM influences treatment decisions.

## Supplementary Material

kead621_Supplementary_Data

## Data Availability

The data underlying this article will be shared upon reasonable request to the corresponding author.
