# Supplementary material for: An international multicentre analysis of current prescribing practices and shared decision-making in psoriatic arthritis
Source: Rheumatology (Oxford). 2023 Nov 27;63(12):3449–56. doi: 10.1093/rheumatology/kead621 (PMC11636566; doi:10.1093/rheumatology/kead621)
Supplement: kead621_Supplementary_Data [file kead621_supplementary_data.docx]

## **Title: An international multi-centre analysis of current prescribing practices and shared decision-making in psoriatic arthritis**

**Supplementary material**

*Supplementary Table S1. Measures of shared decision-making.*

CollaboRATE. Each of the following is scored from 0 (no clinician effort) to 9 (maximum clinician effort):

1. How much effort was made to help you understand your health issues?
2. How much effort was made to listen to what matters most to you about your health issues?
3. How much effort was made to include what matters most to you in choosing what to do next?

PEPPI-5. Each of the following is scored from 1 (no confidence) to 5 (very confident):

1. How confident are you in your ability to know what questions to ask a doctor?
2. To get a doctor to answer all of your questions?
3. To make the most of your visits with your doctors?
4. To get a doctor to take your chief health concern seriously?
5. To get a doctor to do something about your chief health concern?
